# A dataset for successful recognition of cucumber diseases

**DOI:** 10.1016/j.dib.2023.109320

**Published:** 2023-06-16

**Authors:** Nusrat Sultana, Sumaita Binte Shorif, Morium Akter, Mohammad Shorif Uddin

**Affiliations:** Department of Computer Science and Engineering, Jahangirnagar University, Dhaka, Bangladesh

**Keywords:** Image recognition, Agriculture, Cucumber dataset, Deep learning, Computer vision

## Abstract

Plant disease is a common impediment to the productivity of the world's agricultural production, which adversely affects the quality and yield of crops and causes heavy economic losses to farmers. The cucumber is a frequently grown creeping vine plant that has few calories but is high in water and several vital vitamins and minerals. Due to the unfavorable ecological environment and non-biological circumstances, cucumber diseases will adversely harm the quality of cucumber and cause heavy financial loss. Early identification and protection of crop diseases are essential for disease management, crop yield enhancement, cost reduction, and boosting agricultural production. The traditional diagnosis of crop diseases is often time-consuming, laborious, ineffective, and subjective. To cope with this scenario, the development of a machine-based model which can detect cucumber diseases is a demand of time for increasing agricultural production. This article offers a major cucumber dataset to build an effective machine vision-based model which can detect more variety of cucumber diseases. The dataset includes eight different types of classes containing disease-affected and disease-free cucumber images (Anthracnose, Bacterial Wilt, Belly Rot, Downy Mildew, Pythium Fruit Rot, Gummy Stem Blight, Fresh leaves, and Fresh cucumber) which were collected from the 6th to 30th of May 2022 from real fields with the cooperation of an expert from an agricultural institution.

The dataset is hosted by the Department of Computer Science and Engineering, Jahangirnagar University, and is freely accessible at https://data.mendeley.com/datasets/y6d3z6f8z9/1

Specification table

**Table:** 

Subject:	Computer Science
Specific subject area:	Identification of Plant Diseases, Deep Learning, and Computer Vision:
Type of Data:	Image
How the data were acquired:	From 6th to 30th May 2022, we collected disease-free and various disease-affected cucumber images from real fields using Nikon D5300 a Single-lens reflex digital camera to obtain raw images.
Data format:	Raw jpg
Description of data collection:	Previously, no such pre-treatment of the samples was done. With the cooperation of an expert from an agricultural institution, we gathered all the cucumber disease images along with disease free from real fields.
Data source location:	**Institution:** Cultivated cucumber field of size 4356 square feet by a local farmer. **Zone:** Basundia, Jashore **Country:** Bangladesh
Data accessibility:	**Repository name:** Mendeley Data **Data identification number (DOI number):** 10.17632/y6d3z6f8z9.1 **Link of the dataset:**https://data.mendeley.com/datasets/y6d3z6f8z9/1

## Value of the Data

• Cucumber disease detection is typically done manually by individuals, which is time-consuming and ineffective for both farmers and retailers. For this reason, the development of a machine vision-based model is essential that will minimize human effort, expense, and production time in the agricultural industry by identifying different cucumber diseases. An effective dataset is a prerequisite for developing a better classification model.
• This dataset presents the visual appearance of cucumber diseases in a hyperspectral order of cucumber images. Therefore, it allows researchers to identify and categorize cucumber diseases at an early stage by developing an efficient agricultural automation system.
• Observed images can be employed to build, train, test, evaluate, and differentiate different deep-learning models for disease assessment based on numerous characteristics.
• The original images of cucumber diseases are snapped from a broader view along with a greater diversity of disease classes in comparison to other existing datasets [1,2] that work on binary classes (healthy and unhealthy) with a limited number of data. This will assist in the detection of numerous cucumber diseases and improve performance, which will further help plant physiologists in conducting in-depth analyses to develop an automated disease detection system.
• This image was gathered from real fields in natural weather with inconsistent lighting conditions. As a result, diagnosing diseases with the naked eye may be difficult for researchers.

## 1 Objective

The main objective of this article is to provide an extensive cucumber dataset consisting of more disease classes to build an effective machine vision-based recognition model which can automatically recognize various cucumber diseases for increasing agricultural efficiency, boosting production, and ensuring food security.

## 2 Dataset description

The cucumber is a member of the Cucurbitaceae family that is ideal for detoxification and can hold 96% of its weight in water, preventing dehydration. However, cucumber production is decreasing these days due to several diseases that farmers cannot notice with their naked eyes [3]. As a result of their inadequate training and education, they are unable to confer with agricultural specialists when needed. Cucumber disease is a significant factor in lowering cucumber yields and utilization in various agricultural industries. In these situations, this dataset can serve as a state-of-the-art mentor for developing machine vision-based algorithms for the early recognition and classification of cucumber diseases in the agricultural field [4]. To construct machine vision-based algorithms, we offer a comprehensive cucumber dataset containing eight types of cucumber classes, namely Anthracnose, Bacterial Wilt, Belly Rot, Downy Mildew, Pythium Fruit Rot, Gummy Stem Blight, Fresh leaves, and Fresh cucumber. This cucumber dataset contains (160 × 8=) 1280 original images. Table 1 describes the sample images of the eight classes. At first, we identify the target diseases to be included in the dataset. This may involve conducting research to identify common cucumber diseases, consulting with experts in the field, or using existing datasets as a starting point. Once the target diseases have been identified, we collect plant samples that exhibit the desired symptoms.

**Table 1:** Description of eight classes of sample images in the cucumber dataset.

Class Name	Description	Visualization
Anthracnose	Anthracnose is a frequent fungal disease of cucumbers. The fungus Colletotrichum orbiculare is responsible for anthracnose. During the growing period, the disease can trigger fruit rot, stem canker, leaf spots, and a blight on all cucumber portions that are above ground. Heavy infections of the disease during warm, humid summers may cause early defoliation, yield reductions, and poorer fruit quality. The symptoms most frequently seen in fields are leaf spots; they start in damp environments and eventually develop into tiny, circular yellow dots.	
Bacterial Wilt	Bacterial wilt is a usual and disastrous disease of cucumbers. Erwinia tracheiphila, a bacterium, is responsible for cucumber bacterial wilt, which is first characterized by the withering and drying of individual leaves, especially those with cucumber beetle damage. The most prominent indication of bacterial wilt in crops is wilting, which leads to ultimate demise. Initial symptoms can be seen on a single vine's leaves. As the disease worsens, chlorotic and necrotic regions may appear on leaves. Symptoms often appear along individual runners quickly, progressing to the plant's crown and ultimately to the death of the entire plant.	
Belly Rot	Belly rot in fruit is produced by the fungus Rhizoctonia solani. When temperatures are warm and humidity levels are high, the fungus becomes active. Cucumber fruit develops water-soaked, tan to brown lesions as a result of belly rot symptoms that appear on the blossom end and bottom. Lesions swell, craters, take on an uneven shape, and dry out as the disease develops. Fruits that are infected are firm, and soft rot rarely happens.	
Fresh Leaf	Cucumis sativus, an annual warm-season vine plant in the Cucurbitaceae family, is planted for its nutritious cucumber fruit. Fresh cucumber leaves should be a brilliant medium to dark green color, without any yellowing or browning. The surface of the leaves should be smooth and somewhat waxy, and they should feel crisp and hard to the touch. The leaves should be attached to the stem by long, thin stalks, and should be free of any holes, blemishes, or other diseases- or damage-related symptoms on the leaves.	
Fresh Cucumber	Fresh cucumbers should have no fading or browning and be a vibrant medium to dark green color. Small ridges or lumps may also be seen on the skin of some kinds. The shape of the cucumbers should be straight and homogeneous, without any bending or twisting. Additionally, they must be the same size throughout. Fresh cucumbers should have smooth, spotless skin that shows no evidence of wrinkling or yellowing.	
Pythium Fruit Rot	Several fungus-like organisms of the genus Pythium are responsible for the cucumber fruit rot known as Pythium. The symptoms start as water-soaked lesions, brownish that quickly enlarge and turn big, watery, squishy, and rotting. The rot generally appears on the portions of fruit in contact with the soil. Cucumber fruit frequently has a brown to dark green blister evident on it before the fruit becomes watery and decomposes. Rotten tissues can be seen to have white cottony mycelium, especially under humid conditions.	
Gummy Stem Blight	Gummy stem blight is a fungal disease that can affect cucumber plants. Lesions on the leaves that are brown or grayish-brown may look gummy or sticky. The damaged portions of the leaves may become enlarged and merged lesions that turn yellow or brown. The plant's overall growth and productivity could be hampered by leaves that wilt and finally fall off. In extreme circumstances, the illness can make the cucumber plant's stem rot and collapse, killing the plant entirely.	
Downy Mildew	Cucumber plants are susceptible to downy mildew, a common fungal disease, especially in damp or moist environments. On the upper surface of the leaves of these diseased cucumbers, there are spots or blotches that are yellow or light green. a downy-looking growth on the underside of the leaves that is either white or grayish-purple. The damaged portions of the leaves may develop lesions that spread and converge, turning them brown or necrotic. Curled or distorted leaves could eventually dry out and fall off the plant.	

**Fig. 1:**
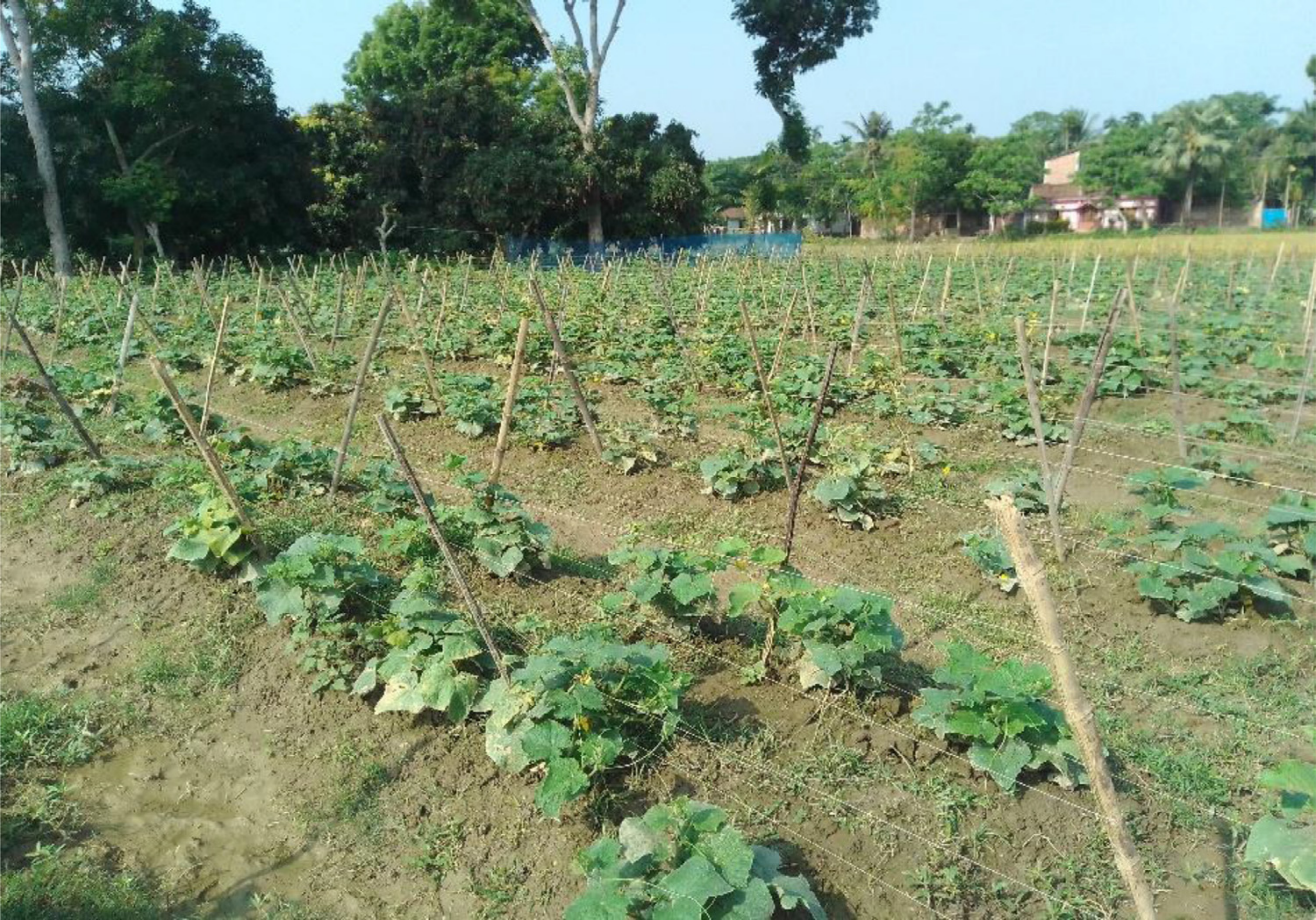
The real cucumber field from where data were collected.

**Fig. 2:**
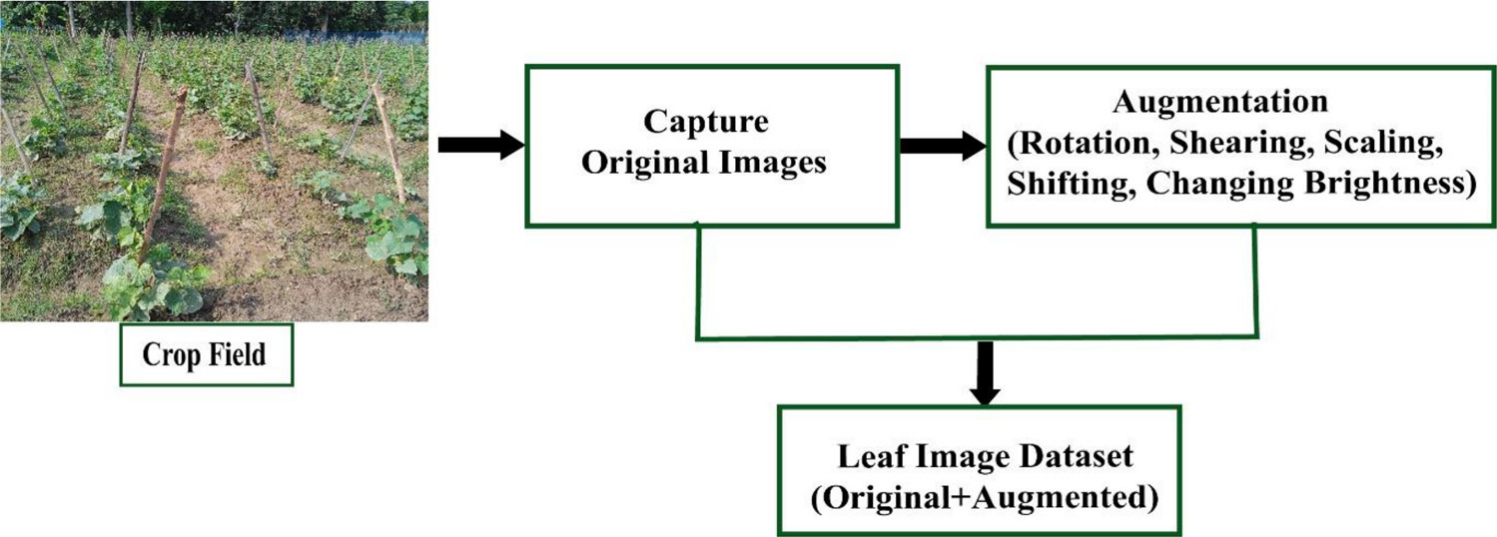
Cucumber disease dataset generation.

**Fig. 3:**
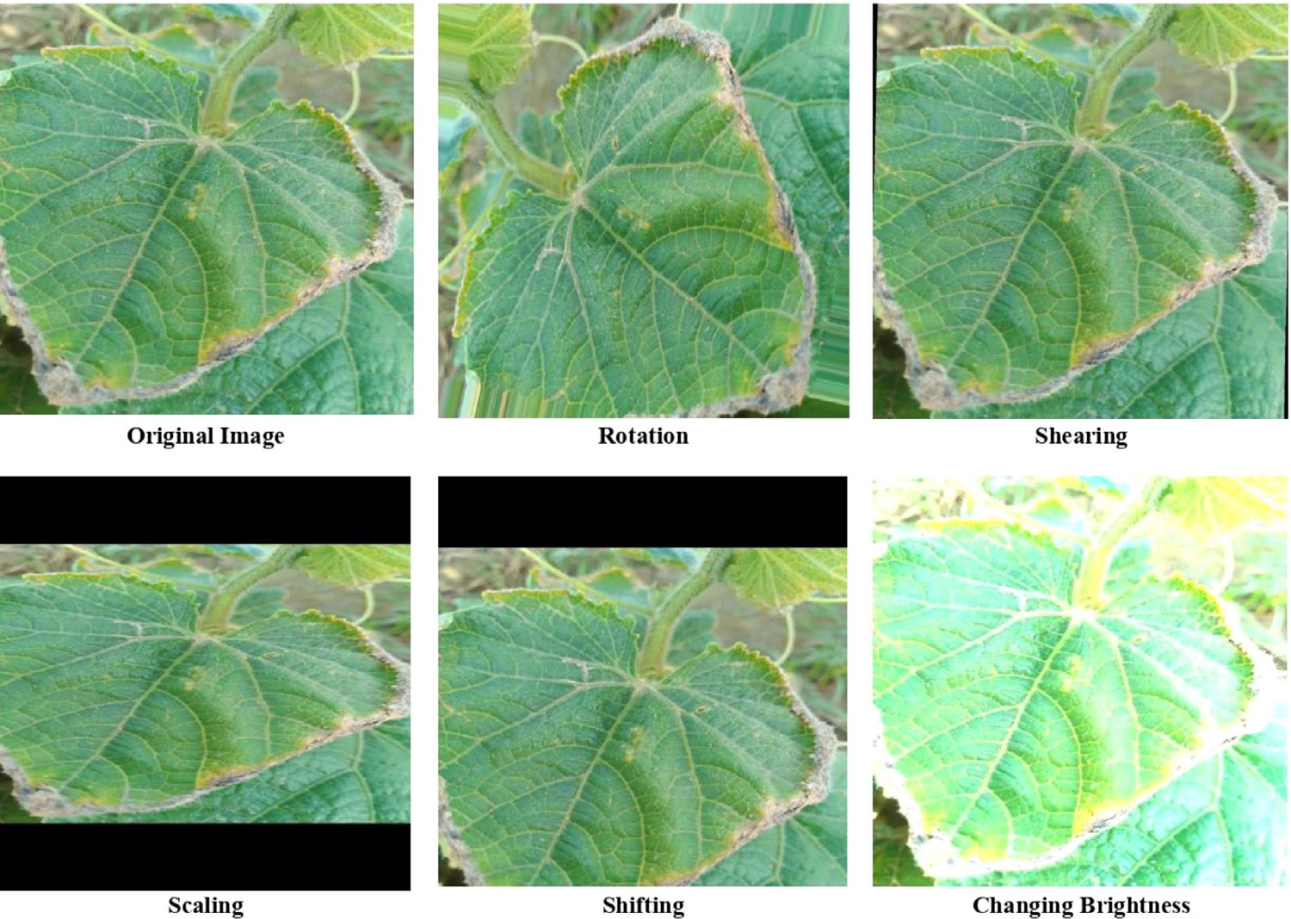
Augmented images.

We captured the images of size 512 × 512 pixels by using a digital camera with the cooperation of an expert from an agricultural institution. The real field from where we collected our data is shown in Fig. 1. After that, we applied data augmentation techniques because machine vision-based deep learning models need a lot of images. Augmentation is performed by using scaling, shifting, shearing, cropping, random rotation, and adjusting brightness [5]. Scaling is done by using bicubic interpolation, bilinear interpolation, and nearest-neighbor interpolation. Brightness is adjusted by using histogram equalization. The fill mode() function from the Keras package in Python is used for estimating parameters. The parameters we used in the augmentation process are: rotation with 45°, 60°, and 90° angles, width shift range, and shear range are 0.2. From each class of 160 original images, we received 800 augmented images. Therefore, the dataset contains a total (800 × 8=)6400 augmented images. Dataset generation is shown in Fig. 2 and augmented images of an original sample image are illustrated in Fig. 3. The statistics of the image dataset are presented in Table 2.

**Table 2:** Statistics of the cucumber dataset.

Category	Number of Original Images	Number of Augmented Images
Anthracnose	160	800
Bacterial Wilt	160	800
Belly Rot	160	800
Downy Mildew	160	800
Pythium Fruit Rot	160	800
Gummy Stem Blight	160	800
Fresh Leaf	160	800
Fresh Cucumber	160	800
**Total**	**1280**	**6400**

The cucumber dataset is available online at the Mendeley repository [6]. This dataset is kept in two folders: one for original images and another for augmented images. Each folder contains 8 subfolders, as there are 8 classes of data named Anthracnose, Bacterial Wilt, Belly Rot, Downy Mildew, Pythium Fruit Rot, Gummy Stem Blight, Fresh leaves, and Fresh cucumber. We kept the two folders as two zip files: Augmented Image.zip and Original Image.zip.

## 3 Experimental design, materials & methods

### 3.1 Camera specification

Nikon D5300 a Single-lens reflex digital camera is used for capturing all the sample images, which image sensor is 23.5 × 15.6 mm CMOS sensor and the Effective pixels are 24.2 million. The Lens mount is a Nikon F mount and The Effective pixels of this camera are 24.2 million and the total camera pixel is 24.78 million. The shutter of this camera is the electronically controlled vertical-travel focal-plane shutter.

### 3.2 Deep learning model validation

**Fig. 4:**
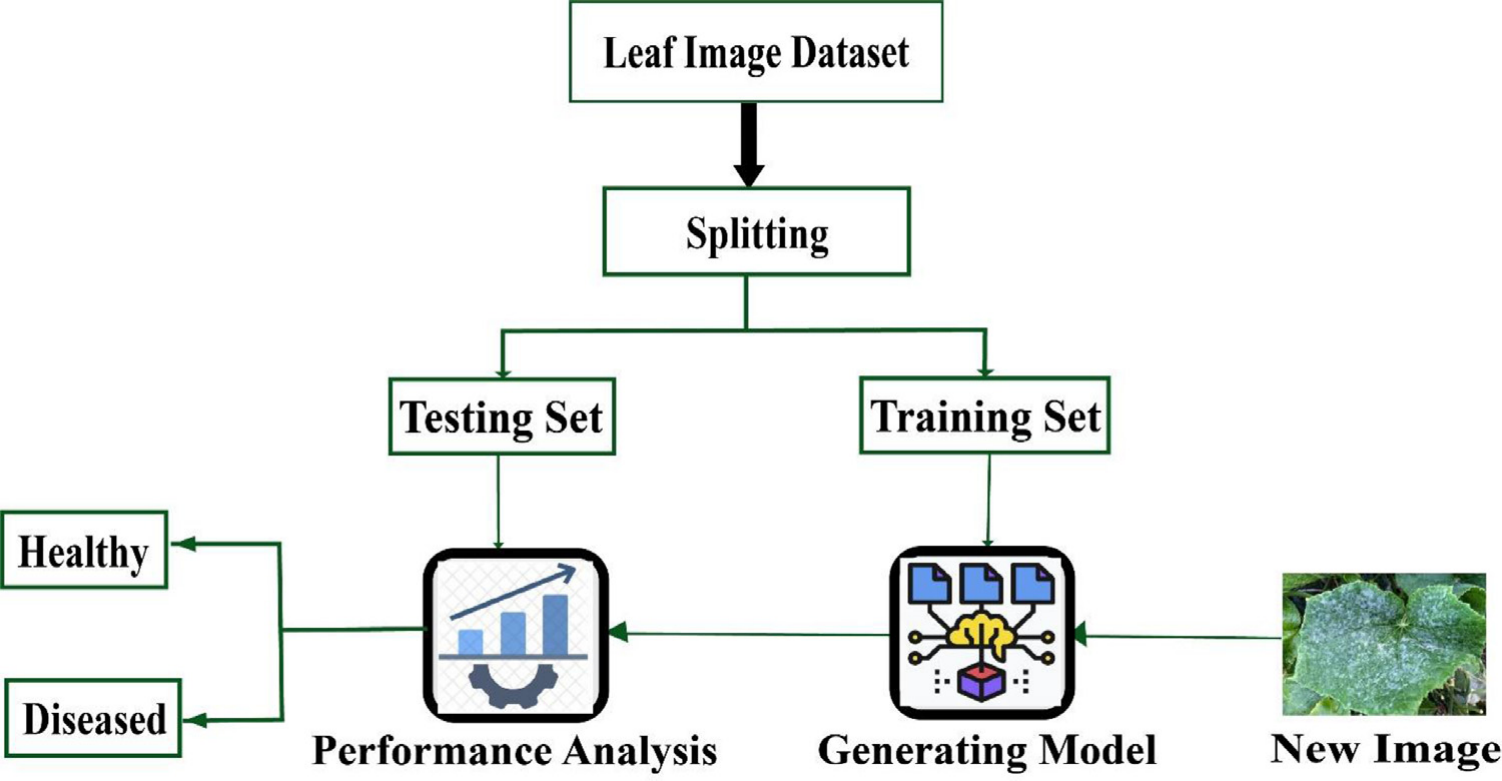
Generic working process for recognition of cucumber diseases.

We offer a conventional deep learning model to effectively train the dataset for producing a state-of-the-art outcome. Validating a deep learning model involves assessing the performance of the model on a dataset. It involves data preprocessing, data splitting, model training, and performance evaluation on a validation set, and model testing on a completely new test set. This process assists in ensuring that the model can generalize to new data and can be relied upon for making accurate results. It is important to preprocess the data to ensure that the model can learn from the data effectively. This may involve scaling, normalization, augmentation, and other data transformations. After that, we split our collected data into two sets - the training set and the testing set. The expected model is trained using the training set, and its performance is assessed using the testing set. The steps to validate a deep learning model on the dataset are illustrated in Fig. 4.

## Ethics statements

This article does not contain any studies with human or animals subjects by any of the authors. The datasets used in the article are open to the public. For the usage of these datasets, proper citation rules should be maintained.

## CRediT author statement

**Nusrat Sultana**: Conceptualization, original draft preparation, Methodology and Data curation. **Sumaita Binte Shorif**: Original draft preparation, Methodology and Software. **Morium Akter**: Visualization and Editing. **Mohammad Shorif Uddin**: Supervision, Reviewing, and Editing.

## Declaration of Competing Interests

The authors declare that they have no conflict of interest from any competing financial interests or personal relationships that could have appeared to influence the work reported in this paper.

## Data Availability

Cucumber Disease Recognition Dataset (Original data) (Mendeley Data).
